# Analysis of Mercury Concentration in Honey from the Point of View of Human Body Exposure

**DOI:** 10.1007/s12011-021-02744-9

**Published:** 2021-06-06

**Authors:** Agnieszka Fischer, Barbara Brodziak-Dopierała, Joanna Bem, Bożena Ahnert

**Affiliations:** Department of Toxicology and Bioanalysis, Faculty of Pharmaceutical Science, Medical University of Silesia, 30 Ostrogórska Str, 41-200 Sosnowiec, Poland

**Keywords:** Honey, Mercury, AAS

## Abstract

Honey is a highly valued product due to its nutritional value, pro-health and healing properties. Pollutants from the environment penetrate into nectar, honeydew, pollen and next into bee products and can cause human exposure after ingestion. Mercury (Hg) is a toxic metal to living organisms. This is why it was important to determine the level of Hg in consumed honey.

The aim of this manuscript is to analyse mercury concentration in honeys collected on the territory of Poland. A total of 108 samples of honey purchased in regional apiaries and hypermarkets were tested. The concentration of Hg was analysed in various types of honey (multifloral, honeydew, linden, goldenrod, acacia, buckwheat, rapeseed, sunflower, heather, dandelion, phacelia). The values of the Estimated Daily Intake (EDI), Estimated Weekly Intake (EWI) and % Provisional Tolerable Weekly Intake (% PTWI) were calculated. This allowed estimating the amount of Hg taken during consumption of the tested honeys.

The concentration of Hg ranged from 0.01 to 1.71 µg/kg and was 0.43 µg/kg on average. A higher concentration of Hg, which was statistically significant, was recorded in honeydew honey, then in compound honeys. Honeys produced from one raw material had the lowest concentration of Hg. There were no significant differences in the concentration of Hg depending on the origin of honey. The calculations have shown that consumption of a portion (19 g) of the tested honey per week is safe for both adults and children according to the applicable standards.

## Introduction

The development of the economy, in particular the industry, has contributed to an increase in the standard of human life, but also in environmental pollution by heavy metals [1]. Bees are exposed to different sources of contaminants through pollen and nectar that contain heavy metals of natural and anthropogenic origin. There are more and more reports of the possible contamination of honey and other bee products [2–5]. Mercury (Hg) is a heavy metal listed among the top 10 contaminants [6]. When in a methylated form, its capacity to penetrate the biological barriers of the human organism is high [7]. It easily crosses the blood–brain barrier through LAT-type amino acid transporters and its high affinity for lipid structures, the effects of which are accumulation in the brain and neurotoxicity [8, 9].

Honey is produced by honeybees (*Apis mellifera* L.) from nectars of flowers, from secretions of plant parts or from excreta of sucking insects [10, 11]. Honey consists of simple sugars, mainly fructose and glucose (75%), disaccharides, mainly sucrose (3–10%), as well as other substances such as amino acids, vitamins (A, B_1_, B_2_, B_6_, B_12_, C), organic and inorganic acids, flavonoids and enzymes [12–14]. The final content of these compounds and honey composition depend on the type of nectar or honeydew that was collected to produce honey, the harvesting season, environmental factors and individual treatments used by beekeepers [15, 16].

Honey has an osmotic effect due to the high sugar content and low water content. It has a pH within the range of 3.2–4.5, which inhibits the development of pathogenic substances, and extends its shelf-life. This is also due to the production of hydrogen peroxide by the enzyme—glucose oxidase [15].

Honey has many valuable nutrients and has healing properties. Pro-health properties result from the contained minerals, microelements and trace elements, and these depend on the type of honey and the place of collection [17].

The most important mineral components of honey are the following oxides: Na, K, Ca and P. The content of minerals in honey (0.1–0.2% in nectar honey, 1% in honeydew) depends on soil conditions on which honey plants grew [14, 18]. Dark types of honey are characterised by a higher content of minerals [14, 19]. Honey contains numerous micronutrients and trace elements: Mg, Si, Pb, Cd, Cr, Al, B, Sn, Ag, Ba, As, Mo, Mn, Co, Se and bioelements such as Fe, Zn and Cu [17, 20–22].

Apart from its nutritional properties, honey has antibacterial, antifungal and antiviral properties. The antibacterial activity depends on the presence of sugar, pH, hydrogen peroxide and phytochemical components of honey [14, 23]. Honey may protect against gastrointestinal infection [14, 23, 24]. It is used to heal wounds, soothe inflammation and ulceration of the skin, and it supports treatment of infections, asthma and respiratory diseases [14, 23, 25, 26]. Studies have shown that topical application of honey can be effective in the treatment of seborrheic dermatitis and prevent hair loss [14, 27]. Honey has an antimutagenic effect—it protects the bladder and breasts [28, 29]. Like propolis, it strengthens the immune system and reduces the risk of viral diseases [28, 30].

There is a close correlation between the accumulation of heavy metals in soil and plants and the content of heavy metals in bee products [31]. Hence, honey can be a useful environmental quality indicator within its collection area, which is about 7 km^2^ [14, 32]. Environmental pollutants can reach bee products from the air, water and soil [33, 34]. Therefore, research on determining the concentration of Hg in honey obtained from bees living in various environmental conditions is important.

The conducted research is the result of the growing interest in natural medicine and natural products of bee origin. As a natural product, honey is subject to the effects of environmental pollutants, including heavy metals. The aim of the study was to analyse the concentration of mercury in honeys purchased in Poland. Honey samples were taken from apiaries located throughout the country and contained variable environmental pollution. Thus, it was determined whether region of origin influences the concentration of Hg in honey. For comparison, the concentration of Hg in honeys purchased in hypermarkets, which did not contain precise information about the place of origin, was also tested. The amount of Hg in honey, depending on type, was tested. To determine the safety of honey consumption and potential human exposure, the values of daily and weekly Hg intake during the consumption of the tested honeys were calculated, % Provisional Tolerable Weekly Intake (% PTWI), and these values were referred to the acceptable standards.

## Material and Methods

The subject of the research was 108 honey samples purchased in Poland in 2018–2020. The study analysed 11 types of honey (multifloral, honeydew, linden, goldenrod, acacia, buckwheat, rapeseed, sunflower, heather, dandelion, phacelia).

### Sampling

**Fig. 1:**
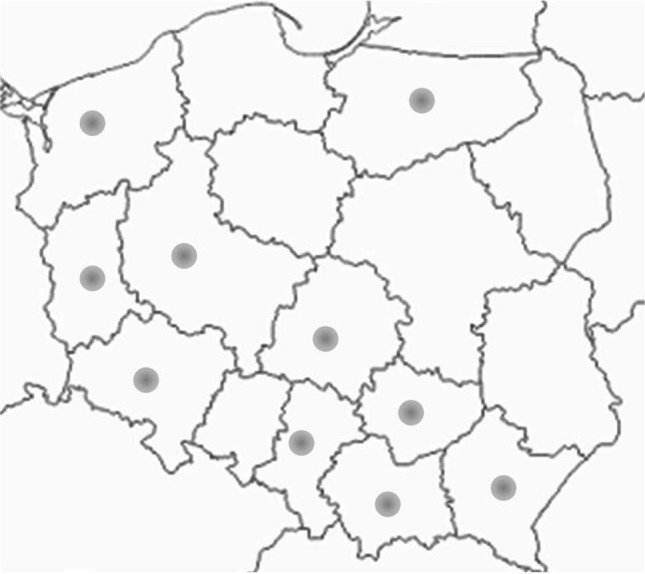
The area of Poland divided into provinces with the location of apiaries. ●The location of the apiaries where the honey for research was collected

The honey was purchased from individual sellers (regional apiaries) (Fig. 1) and in hypermarkets. Information on honey was read from the labels placed on the packaging. According to the information, the honey was obtained from various provinces covering the entire territory of Poland; it was also prepared by producers from blends of honeys from the territory of the European Union and from countries not in the European Union.

### Determination

The concentration of Hg was determined in the honey samples using the AMA 254 analyser (Altec, Czech Republic). Measurement conditions were wavelength—253.65 nm, carrier gas—technical oxygen and inlet pressure—200–250 kPa. Specific intervals for the analysis process were used (s): 120 (drying), 140 (decomposition) and 60 (detection) [17, 18]. The limit of detection (LOD) = 0.001 ng Hg; limit of quantitation (LOQ) = 0.005 ng Hg. The measurement concerned the total amount of Hg, regardless of its form in the sample. The AMA 254 analyser does not require sample preparation before determination. The samples for analysis (average weight = 0.10393 g) were weighed on an analytical balance (RADWAG, Poland). Three independent test samples were prepared from each honey. The concentration of Hg in the honey sample was the arithmetic mean of 3 measurements.

Validation of designations was checked using reference material (INCT-MPH-2 Mixed Polish Herbs). The Hg concentration in reference material, measured: *n* = 5, was 0.0154–0.0175 µg Hg/g (mean 0.0166 ± 0.0001 µg Hg/g).

### Statistical Analysis

The concentration of Hg in honey samples was made using the Statistica ver. 13 (StatSoft, Poland). The normality of the distribution was checked using the Shapiro–Wilk test. As the concentration of Hg in the analysed samples differed from a normal distribution, the statistical significance and its level were checked with the Mann–Whitney U test (for two samples) and the Kruskal–Wallis H test (for a greater number of samples). The value of *p* < 0.05 was considered statistically significant.

### Risk Calculations

The safety of honey consumption by children and adults was analysed on the basis of the % Provisional Tolerable Weekly Intake (% PTWI) value, which was calculated in relation to the acceptable level [35]. These values for adults are 1 µg/kg bw per week and for children 4 µg/kg bw per week. The average daily honey consumption was set at 19 g [36, 37]. The average weight for adults was 70 kg and for children—15 kg. The Estimated Daily Intake (EDI) and the Estimated Weekly Intake (EWI) of Hg and % Provisional Tolerable Weekly Intake (% PTWI) were calculated using the following formulas, in accordance with [37, 38]:

$$\mathrm{EDI}=\frac{\mathrm{mean}\ \mathrm{daily}\ \mathrm{consumption}\ \mathrm{of}\ \mathrm{honey}\times \mathrm{metal}\ \mathrm{level}}{\mathrm{body}\ \mathrm{weight}}$$

where:

EDI—Estimated Daily Intake

EWI—Estimated Daily Intake × 7

EWI—Estimated Weekly Intake

$$\%\mathrm{PTWI}=\frac{\mathrm{Estimated}\ \mathrm{Weekly}\ \mathrm{Intake}\ \mathrm{Cd}\times 100\ }{\mathrm{PTWI}}$$

where % PTWI—% Provisional Tolerable Weekly Intake.

## Results

The statistical analysis of concentration of Hg in samples of various types of honey is presented in Table 1. The concentration of Hg in the tested honeys ranged from 0.01 to 1.71 µg/kg and was 0.43 µg/kg on average. The coefficient of variation amounting to 96.4% proves that there is a high variability of Hg concentration in the tested samples. The greatest number of honeys (*n* = 30) was the multifloral type. The average concentration of Hg in multifloral honey (median 0.33 µg/kg) was characterised by a high coefficient of variation (98.2%). The highest maximum concentration of Hg among all analysed types of honey was found in multifloral honey (1.70 µg/kg). The sample with the greatest concentration of Hg was purchased in the apiary of a local producer from the province of Lesser Poland (Southern Poland). The most similar results of the concentration of Hg in the samples were found for phacelia honey (coefficient of variation—51.0%). In relation to the type of honey, the highest average concentration of Hg was recorded in honeydew honeys (median 0.71 µg/kg), the lowest in dandelion honeys (median—0.12 µg/kg), but the number of samples of this type was limited (*n* = 2). The relatively low concentration of Hg was also recorded in buckwheat honey (*n* = 15, median 0.15 µg/kg) and goldenrod honey (*n* = 5, median 0.09 µg/kg). The honeys, depending on the type and value of the median of Hg (increasing), are as follows: goldenrod, dandelion, buckwheat, rapeseed, sunflower, heather, linden, multifloral, acacia, phacelia and honeydew. LOQ of Hg in various types of honey (multifloral, honeydew, linden, goldenrod, acacia, buckwheat, rapeseed, sunflower, heather, dandelion, phacelia) was not statistically significant (*p* = 0.18, Kruskal–Wallis H test).

**Table 1:** Statistical analysis of the concentration of Hg in various types of honey (µg/kg)

Type of honey	*n*	AM	Range	SD	Me	Percentile				CV%
						10	25	75	90	
All honey	108	0.43	0.01–1.71	0.41	0.31	0.06	0.13	0.60	1.08	96.4
Acacia	10	0.41	0.01–1.03	0.32	0.35	0.03	0.18	0.69	0.88	79.4
Phacelia	4	0.36	0.11–0.52	0.18	0.41	0.11	0.23	0.49	0.52	51.0
Buckwheat	15	0.28	0.07–1.11	0.28	0.15	0.07	0.10	0.35	0.62	99.7
Linden	15	0.35	0.01–0.97	0.28	0.31	0.01	0.10	0.55	0.70	80.3
Dandelion	2	0.12	0.03–0.22	0.13	0.12	0.03	0.03	0.22	0.22	107
Goldenrod	5	0.36	0.02–1.30	0.54	0.09	0.02	0.04	0.34	1.30	152
Rapeseed	10	0.25	0.01–0.71	0.20	0.22	0.02	0.16	0.32	0.56	81.2
Sunflower	2	0.27	0.10–0.44	0.24	0.27	0.10	0.10	0.44	0.44	89.7
Honeydew	13	0.72	0.07–1.55	0.46	0.71	0.11	0.36	0.93	1.30	64.3
Multifloral	30	0.54	0.06–1.71	0.53	0.33	0.08	0.17	0.74	1.55	98.2
Heather	2	0.29	0.11–0.47	0.26	0.29	0.11	0.11	0.47	0.47	89.4

*AM*, arithmetic mean; *SD*, standard deviation; *Me*, median; *CV*, coefficient of variation

**Fig. 2:**
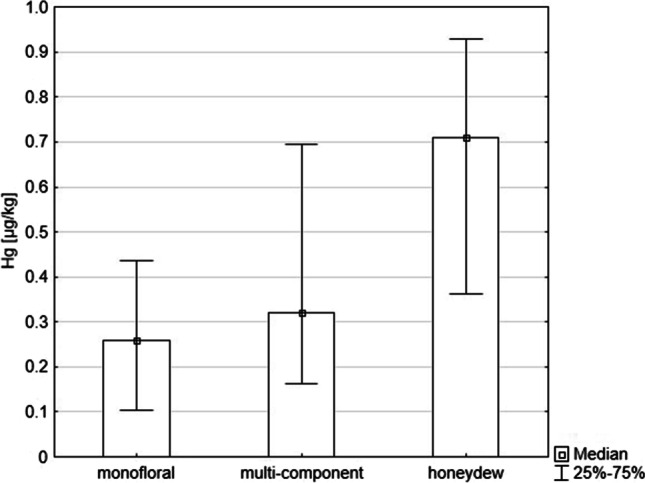
Comparison of the concentration of Hg in single-component (monofloral), multi-component (polyfloral) and honeydew honeys

The tested honey samples were grouped according to their composition into multi-component, single-component and honeydew honey. Statistically significant differences were found in the concentration of Hg depending on the composition of honey (single-component, multi-component and honeydew) (*p* = 0.01, Kruskal–Wallis H test). The highest concentration of Hg was found in honeydew honeys (median 0.71 µg/kg)—more than twice as high as in multi-component honeys (median 0.32 µg/kg) and three times higher than in single-component honeys (median 0.26 µg/kg) (Fig. 2).

**Fig. 3:**
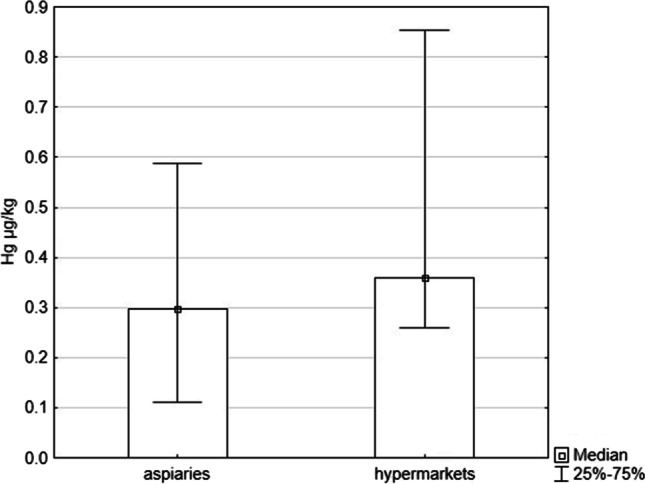
Comparison of the concentration of Hg in honeys bought in hypermarkets and apiaries

**Fig. 4:**
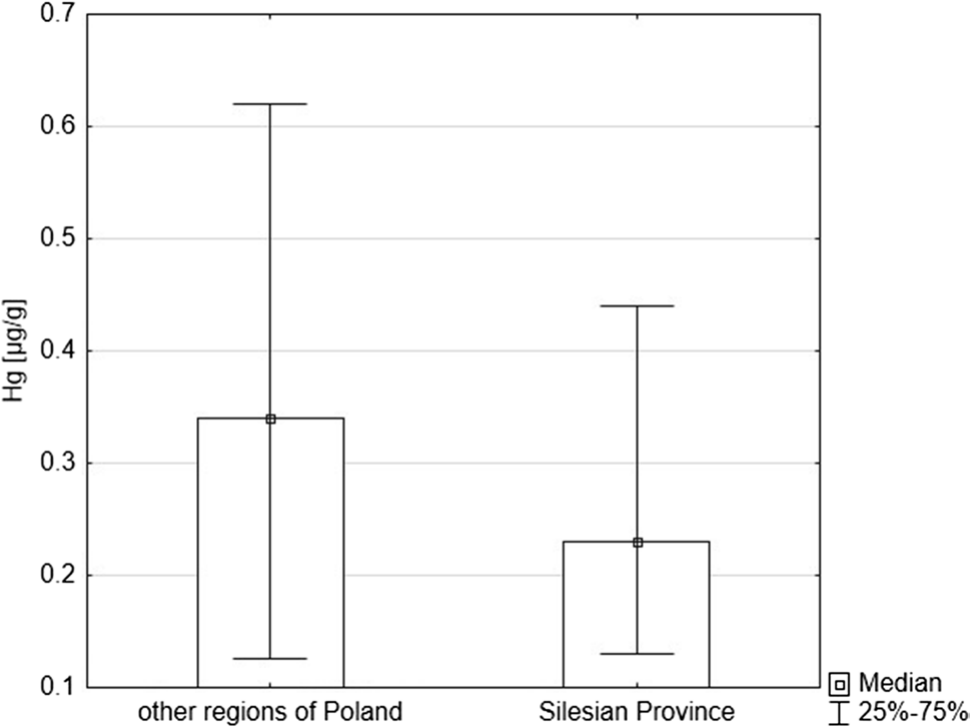
Comparison of the concentration of Hg in honeys from the Silesian Province* and other regions of Poland. *Silesian Province is the most industrialized and highly urbanized region of Poland

The analysed honeys were purchased in hypermarkets and apiaries. The average concentration of Hg was higher in honeys purchased in hypermarkets than in regional apiaries (median, respectively: 0.30 µg/kg and 0.36 µg/kg); these differences were not statistically significant (*p* = 0.12, Mann–Whitney U test) (Fig. 3). The honeys purchased in stores contained information that their place of origin was the EU and non-EU countries. The measurement of the concentration of Hg in honey depending on the location in Poland did not show statistically significant differences as well. Honey samples from the Silesian Province, which is the most industrialised and highly urbanised region of Poland, had a lower Hg concentration than in other regions (Fig. 4) (*p* = 0.877, Mann–Whitney U test).

Table 2 shows the calculated values of EDI, EWI and % PTWI. The consumption of 19 g of honey per week is safe for human health, as it results in Hg intake at the level of 0.009–0.020% PTWI for adults and 0.010–0.023% PTWI for children.

**Table 2:** Safety of honey consumption in terms of mercury presence (mean Hg concentration and max Hg concentration)

Type of honey	*n*	Mean Hg (µg/kg)	Adults			Children			Max Hg (µg/kg)	Adults			Children		
			EDI	EWI	% PTWI	EDI	EWI	% PTWI		EDI	EWI	% PTWI	EDI	EWI	% PTWI
Single-component	60	0.32	1.22E − 05	8.56E − 05	0.009	5.71E − 05	4.00E − 04	0.010	1.30	5.03E − 05	3.52E − 04	0.035	2.35E − 04	1.64E − 03	0.041
Multi-component	35	0.51	1.96E − 05	1.37E − 04	0.014	9.17E − 05	6.42E − 04	0.016	1.71	6.63E − 05	4.64E − 04	0.046	3.09E − 04	2.17E − 03	0.054
Honeydew	13	0.72	2.79E − 05	1.96E − 04	0.020	1.30E − 04	9.13E − 04	0.023	1.55	5.99E − 05	4.19E − 04	0.042	2.80E − 04	1.96E − 03	0.049
All honey	108	0.43	1.67E − 05	1.17E − 04	0.012	7.78E − 05	5.45E − 04	0.014	1.71	6.63E − 05	4.64E − 04	0.046	3.09E − 04	2.17E − 03	0.054

*PTWI*, Provisional Tolerable Weekly Intake, *% PTWI*, % Provisional Tolerable Weekly Intake, *EDI*, Estimated Daily Intake, *EWI*, Estimated Weekly Intake

PTWI 1 µg/kg bw per week for adults and 4 µg/kg bw per week for children [31]

## Discussion

As a component of a daily diet, natural products are now more and more valued and desired. There is also a belief that natural products are healthy and safe. Honey has a pro-health effect that is used in natural medicine and is also valued for its taste. It can be used as an auxiliary in the treatment of many diseases, including the upper respiratory tract, cardiovascular system, gastrointestinal tract and urinary system [23–30]. The effect of honey depends on the raw material from which it is made. The most important effects were anti-inflammatory, antibacterial, antiviral and antifungal. What is more, honey is easily available, both in grocery stores, herbal stores, pharmacies or directly in apiaries. Despite its beneficial effects, honey may contain environmental pollutants, e.g. heavy metals, including Hg. Mercury is absorbed by plants along with water and minerals. Pollen and honeydew obtained from plants are used by bees to produce honey, which is why bee products may contain Hg compounds.

The maximum concentration of Hg in honey specified in the EU Commission Regulation 2018/73 of 16 January 2018 that amended Annexes II and III to Regulation (EC) No 396/2005 of the European Parliament and of the Council is 0.01 mg/kg [39]. The average concentration of Hg determined in the tested honeys (0.43 µg/kg) did not exceed the acceptable standards in any of the analysed samples. Also, the maximum concentration of Hg determined in the honey samples (1.71 µg/kg) did not exceed the above-mentioned normative values.

Madras-Majewska et al. conducted similar research in Poland [40]. The concentration of Hg in honeys from the apiaries was 0.27 µg/kg and was similar to the values obtained in our own research. In the studies by Dżugan et al. [41], carried out in Poland as well, but covering only a selected area (South-Eastern Poland, Sub-Carpathian Province), the concentration of Hg in honey and in organisms of bees, determined using the ICP-OES method, was below the limit of quantification (< 1 µg/g).

In the research from the territory of Italy (8 provinces) [10], the concentration of Hg was at a similar level as in the authors’ own research. The average concentration of Hg was 0.19 µg/kg, and the range of changes was 0.04–1.46 µg/kg, similar to our research (0.01–1.71 µg/kg). Other research from the same country showed that the concentration of Hg was 0.007 µg/g [42], which was much lower.

The analysis of honey for Hg concentration in another European country, the Czech Republic, showed slightly higher values in relation to our own research: 0.67–2.93 µg/kg [43].

Toth et al. [44] studied the content of Hg in honeys from eastern Slovakia. Honey from the city of Košice had 0.081 µg Hg/kg, and honey from the rural areas of Rozhanovce had 0.079 µg Hg/kg. No statistically significant differences were found between the concentration of Hg in honeys from urban and rural areas.

A comparison of Hg content in honeys from rural and urban areas was also made by Maggid et al. [33]. The average level of Hg in rural honeys in Tanzania was 11.910 µg/kg and from London—7.023 µg/kg. A reverse relation was described by Toporcák et al. [45] when examining honey samples from Slovakia. The concentration of Hg for honeys obtained from polluted areas was in the range of 50–212 µg/kg, while from non-polluted areas it was many times lower (1–3 µg/kg). Our research showed no statistically significant differences in the concentration of Hg depending on the origin of honey. Moreover, honeys from the industrial region (Silesian Province) had a lower concentration of Hg than from other areas in Poland.

The comparison of the results based on literature data shows a different level of Hg concentration in honeys. These differences are clearly visible in relation to the geographical origin of honey in different countries.

Honeys from Greece had the Hg concentration lower than 50 µg/kg [46], similar to the research by Toporcák et al. [45] from industrial areas in Slovakia, where these values were much higher than in the authors’ own research. Similar results were in the studies by Akbari et al. [47] in honey from Iran, where the Hg concentration was as high as 3.03 mg/kg. The studies by Bilandzic et al. [13] conducted in Croatia showed that the average Hg concentration was higher—2.72 µg/kg.

The research by Jovetić et al. [48] determined the concentration of Hg in honeys from the city of Belgrade. The obtained results were significantly higher than in the authors’ own research and ranged between 73 and 519 µg/kg [48]. Scientists explain that the increased level of Hg is due to anthropogenic sources of air pollution, especially combustion of fossil fuels, which are still used for heating in many houses in the Zemun district of Belgrade [48].

What is more, a high average Hg concentration in honey in relation to our research was found in Nigeria [49], Libya [50] and Malaysia [51]. However, the concentration of Hg in honey, researched by Indian scientists, was determined to be below the apparatus’ detection level [52].

Our research included a comparison of the concentration of Hg in honeys from different places of purchase. Although, the concentration of Hg in honeys from hypermarkets was higher than in products purchased directly from producers (in apiaries), these differences were not statistically significant. This analysis also included a product comparison in terms of the origin of ingredients. Honey bought in hypermarkets was generally defined by producers (without specified place of origin) as a mixture of honeys from EU and non-EU countries. In this case, there were no statistically significant differences in the concentration of Hg in honey as well.

In the case of Hg concentration in different types of honey, reference can be made to the studies by Ru et al. [53] from China. Although, the results of the concentration of Hg in these studies were significantly different from ours (they were several times higher), they indicate that the concentration of Hg may vary depending on the plant raw material from which honey is produced. For comparison, the average Hg concentration in acacia honey was 2.51 µg/kg, in linden honey—4.00 µg/kg and in multifloral honey—2.23 µg/kg [53] (respectively in our research: 0.41, 0.35 and 0.54 µg Hg/kg). The highest concentration of Hg was found in linden honey (4.00 µg/kg) and the lowest in honey obtained from Chinese jujube (0.34 µg/kg) [53]. In our research, honeydew honey contained the highest amounts of Hg (0.72 µg/kg) and dandelion honey the lowest (0.12 µg/kg).

Research of different types of honey from the Koprivnica-Kriżevci agricultural region in Croatia [54] showed that multifloral honeys had a statistically significant (*p* < 0.01) content of Hg (1.35 mg/kg) which acacia honeys did not (0.46 mg/kg). Similarly, in our research, the concentration of Hg was statistically much higher in multi-component honeys than in single-component honeys.

Whereas, in the honey obtained by Tariba Lovaković et al. [55] in Southern Croatia, significantly lower Hg values were found compared to the agricultural region of Koprivnica-Kriżevci. In this research, the average content of Hg in linden honey was comparable to our research and amounted to 0.33 µg/kg, slightly lower in acacia honey—0.34 µg/kg and in sunflower honey slightly higher—0.43 µg/kg (in our research—0.27 µg/kg) [55]. For comparison, the average concentration of Hg in fir honeydew honey was 1.38 µg/kg and in oak honeydew honey—0.99 µg/kg [55] and was higher than in the honeydew honey we tested—0.72 µg/kg.

Differences in the concentration of Hg in particular types of honey we examined were not statistically significant (*p* > 0.05). The highest concentration of Hg was found in honeydew and multi-component honey. However, it was shown that the concentration of Hg in the samples of honeys obtained from one plant (monofloral) was statistically significantly lower than that of multi-component honeys. Similar results were found in the studies by Winiarska-Mieczan et al. [37] where multi-component honeys contained higher amounts of heavy metals (Cd and Pb) than single-component honeys.

Based on the obtained results, the amount of Hg ingested into the body during daily and weekly honey consumption was calculated. The assumption was a weekly consumption of 19 g of honey [36]. The tested honey samples provide the human body with an amount of Hg which is 0.012% PTWI in adults and 0.014% PTWI in children. Thus, the permissible norms of Hg consumption were not exceeded in any of the tested honeys [35]. The mean weekly consumption of Hg ranged from 1.17 to 9.13E − 04 µg/bw/week. In the research by Maggid et al. [33], the weekly consumption of Hg found in honey was much higher (15.98 µg/person/week); however, in our research the average consumption of honey was 19 g per week, and in the research by Maggid et al. [33] it was 316 g. For comparison, EWI values resulting from the consumption of honey from the territory of Poland, calculated for Cd and Pb, were also low—6.91E − 06 and 5.32E − 05 mg, respectively [37].

Summing the obtained results up, none of the tested honeys was found to contain Hg exceeding the permissible value. However, the dynamic development of the industry creates a certain risk. The amount of contamination in honey and other products produced by bees may increase. For example, Madras-Majewska et al. [40] examined the concentration of Hg in bee pollen from various regions of Poland. The range of changes was 0.18–7.59 µg Hg/kg, and the highest concentration of Hg was found in pollen from Northern Poland (West Pomeranian Province—2.28 µg/kg). To ensure the appropriate quality of bee products, which are often used for medicinal purposes, it is advisable to conduct tests for the concentration of Hg and other heavy metals.

## Conclusions

The concentration of Hg in the tested honeys bought in Poland varied greatly. Depending on the type of honey, the concentration of Hg increased as follows: goldenrod, dandelion, buckwheat, rapeseed, sunflower, heather, linden, multifloral, acacia, phacelia and honeydew. Differences in the concentration of Hg, depending on the type of honey, were not statistically significant. Whereas, the comparison of honeydew, multi-component and single-component honeys showed a statistically significant higher concentration of Hg in honeydew and multi-component honeys. The lowest concentration of Hg was found in single-component honeys. The origin of product used to produce honey did not statistically affect the concentration of Hg in the tested honey samples. Honey from apiaries located in Poland did not differ statistically in terms of mercury content. Products purchased in apiaries and hypermarkets did not differ statistically in terms of the concentration of Hg. The calculated values of EDI, EWI and % PTWI have shown that consumption of a portion (19 g) of the tested honey per week is safe for both adults and children according to the applicable standards. This concerns the indices calculated for both the average and the maximum concentration of Hg.
